# Editorial Comment: Sexual Dysfunction in Parkinson Disease: A Multicenter Italian Cross-sectional Study on a Still Overlooked Problem

**DOI:** 10.1590/S1677-5538.IBJU.2021.05.02

**Published:** 2021-07-23

**Authors:** Valter Javaroni

**Affiliations:** 1 Hospital Federal do Andaraí Departamento de Andrologia Rio de JaneiroRJ Brasil Departamento de Andrologia, Hospital Federal do Andaraí, Rio de Janeiro, RJ, Brasil

Loredana Raciti^1^, Maria Cristina De Cola^1^, Paola Ortelli^2^, Francesco Corallo^1^, Viviana Lo Buono^1^, Elisabetta Morini^1^, et al.

J Sex Med. 2020 Oct;17(10):1914-1925.

## COMMENT

In this Italian multicenter observational study, more than 200 patients with Parkinson's disease were evaluated with The International Index of Erectile Function and the Female Sexual Function identifying loss of libido in 68% of men and 53% of women. Overall, 57% of patients reported that Parkinson's disease impacted their sexual life and desire and stated that it affected especially due to reduced sexual desire and the frequency of sexual intercourses. Men were more likely to be affected than women. Authors advice clinicians dealing with PD and other chronic illness to pay more attention to sexual issues.

Parkinson's disease (PD) is a complex disorder with motor impairment (rigidity, tremor, bradykinesia, and postural instability) and nonmotor manifestations/symptoms (NMS) - which can precede the motor symptoms - that result in progressive disability and severe complications, factors that have a significant impact on patients’ quality of life (QoL) (1, 2).

The importance of NMS - such as depression and cognitive impairment - and gender differences in influencing quality of life, as well as progression, institutionalization at advanced disease stage and therapeutic response of Parkinson's disease patients gained more relevance in recent years (3, 4). Mood, NMS burden, and gait problems seem to be the most important factors affecting health-related and global perceived QoL in non-demented PD patients (5, 6).

The NMS of PD include neuropsychiatric disorders, sleep disorders, sensory symptoms, and autonomic disorders. Bladder, bowel, and sexual dysfunction (also called “pelvic organ” dysfunctions) are some of the most common autonomic disorders (7). Among the NMS, urinary and sexual dysfunctions are common and potentially treatable, that is why Urologists could play an important role in such area (8, 9).

The pathophysiology of NMS is still poorly understood, and a dysfunction of both dopaminergic and nondopaminergic systems contributes to their development (10).

Lower urinary tract symptoms (LUTS) in PD result from failure of the basal ganglia to suppress micturition. It has been theorized that the loss of basal ganglia output reduces cortical inhibition of the micturition reflex, leading to detrusor hyperactivity and excessive detrusor contractions, which underlie the symptom of urinary urgency (11). Guidelines advocate the empirical use of anticholinergic medication (with caution with associated fractures, delirium, and cognitive decline) in PD and bladder training (BT) can improve bladder control and continence (11, 12).

Compared with the general population, sexual dysfunction is more common in patients with PD, and, similarly to the general population, it is likely multifactorial (13). This symptom is frequently a result of autonomic dysfunction, but motor impairment, psychological and cognitive disturbances, sleep disorders, medications and changes in appearance are also other possible causes (14). Despite the high frequency and the disabling effect of sexual dysfunction in PD, it is still one of the most poorly investigated aspects of the disease (15). Sexual problems are often under-recognized and undertreated (16).

In the large PRIAMO (17) cohort, sexual dysfunction was present in 19.6%. In men, erectile dysfunction (ED) and premature ejaculation were the most frequent problems. Women with PD, when compared with aged-matched controls, are more likely to endorse vaginal tightness, loss of lubrication, involuntary urination, anxiety, and inhibition. Singer and colleagues found that 60% of men with PD reported ED, compared with 37.5% of age-matched controls. Both men and women may endorse decreased libido (18). Increased libido has been reported as an adverse reaction to levodopa (19), since compulsive sexual behavior can be a manifestation of impulse control disorder induced by dopamine agonists. Weintraub and colleagues (20) reported that 3.5% of patients with PD using a dopamine agonist developed this side effect. Because patients are often not forthcoming regarding sexual symptoms, including hypersexuality as a manifestation of ICD and sexual dysfunction in general, practitioners are encouraged to ask all patients with PD about sexual dysfunction.

Erectile dysfunction, premature ejaculation, and orgasmic dysfunction are the frequent complaints in men with PD (21). Prominent SD complaints in women with PD include low sexual desire, arousal dysfunction, and orgasmic dysfunction.10 On the other hand, in a large cohort study, men with erectile dysfunction were found to be 3.8 times more likely to develop PD compared with men with normal erectile function (22).

It is important to deal with differences in clinical manifestations of PD between males and females. In general, females with PD were significantly worse in psychological features such as anxiety and depression, nutritional status and specific domains of QoL, namely, mobility, emotional wellbeing, social support and bodily discomfort. On the other hand, male PD patients had better nutritional status (though with rather small effect size for difference) and activities of daily living but also more severe orthostasis. These aspects of sexual dimorphism in PD also enlighten the features that are more likely to be affected in each sex and should be specifically targeted when managing male and female individuals with PD (23).

In a recent meta-analysis enrolling 30,150 subjects from both the PD group and healthy control group to determine the relative risk for the association between PD and the risk of developing sexual dysfunction, evidence revealed that PD was associated with an elevated risk of sexual dysfunction in males (7 studies; 1.79; 95% CI). However, when restricted to female subjects, the combined relative risk from 3 eligible studies suggested a lack of significant association between PD and SD (24).

Therefore, sexual manifestations in PD patients vary and deserve urological attention and it is doctor's attribution to ask and open conversation about such relevant aspect of Qol. There are several instruments to be used in clinical trials and institutional protocols like the first largely comprehensive, self-completed NMS questionnaire for PD has been developed and validated (25). It considers 30 items distributed in nine different domains: gastrointestinal, urinary, memory, hallucinations, depression/anxiety, sexual function, cardiovascular, sleep disorder, and miscellany.

And after identifying the sexual dysfunction, it could be treated. And there are some aspects, for example, in prescribing PDE 5 inhibitors for PD men. In the early stages, authors recommend measurement of lying and standing blood pressure before prescribing sildenafil to men with parkinsonism (26). Furthermore, such patients should be made aware of seeking medical advice if they develop symptoms on treatment suggestive of orthostatic hypotension.

Raffaele and colleagues reported improved erection in 84.8% of 33 patients receiving 50 mg of sildenafil daily (27). Other PDE5 inhibitors, such as tadalafil and vardenafil, also seem effective but lack formal studies in PD. These medications, however, should be avoided in patients with hypotension.

We can conclude with the word of researchers Bronner and Korczyn (28): “The longitudinal nature of treating neurologic patients puts physicians in an important position to introduce sexual issues, to assess patients, and to plan interventions and the follow-up needed to ensure that sexual difficulties are resolved. Physicians should proactively initiate “sex talk” with their patients and choose the extent to which an intervention corresponds to their capabilities and time constraints”.
